# A Semiautomatic Multi-Label Color Image Segmentation Coupling Dirichlet Problem and Colour Distances

**DOI:** 10.3390/jimaging7100208

**Published:** 2021-10-07

**Authors:** Giacomo Aletti, Alessandro Benfenati, Giovanni Naldi

**Affiliations:** Environmental Science and Policy Department, Università degli Studi di Milano, 20133 Milan, Italy; giacomo.aletti@unimi.it (G.A.); giovanni.naldi@unimi.it (G.N.)

**Keywords:** image segmentation, random walks, graph theory, colour distance

## Abstract

Image segmentation is an essential but critical component in low level vision, image analysis, pattern recognition, and now in robotic systems. In addition, it is one of the most challenging tasks in image processing and determines the quality of the final results of the image analysis. Colour based segmentation could hence offer more significant extraction of information as compared to intensity or texture based segmentation. In this work, we propose a new local or global method for multi-label segmentation that combines a random walk based model with a direct label assignment computed using a suitable colour distance. Our approach is a semi-automatic image segmentation technique, since it requires user interaction for the initialisation of the segmentation process. The random walk part involves a combinatorial Dirichlet problem for a weighted graph, where the nodes are the pixel of the image, and the positive weights are related to the distances between pixels: in this work we propose a novel colour distance for computing such weights. In the random walker model we assign to each pixel of the image a probability quantifying the likelihood that the node belongs to some subregion. The computation of the colour distance is pursued by employing the coordinates in a colour space (e.g., RGB, XYZ, YCbCr) of a pixel and of the ones in its neighbourhood (e.g., in a 8–neighbourhood). The segmentation process is, therefore, reduced to an optimisation problem coupling the probabilities from the random walker approach, and the similarity with respect the labelled pixels. A further investigation involves an adaptive preprocess strategy using a regression tree for learning suitable weights to be used in the computation of the colour distance. We discuss the properties of the new method also by comparing with standard random walk and k−means approaches. The experimental results carried on the White Blood Cell (WBC) dataset and GrabCut datasets show the remarkable performance of the proposed method in comparison with state-of-the-art methods, such as normalised random walk and normalised lazy random walk, with respect to segmentation quality and computational time. Moreover, it reveals to be very robust with respect to the presence of noise and to the choice of the colourspace.

## 1. Introduction

Splitting an image into non-overlapping sets of pixels is the purpose of image segmentation. The resulting sets, called regions (or segments or objects), are defined based on visual properties extracted by local features. The pixels within a region are required to possess some specified properties of homogeneity or similarity [1]. The typical classification consists in dividing segmentation algorithms as follows: pixel-based algorithms, when individual pixel values form the only information used to perform segmentation; edge-based algorithms, when segmentation is based on the detection of the edges present within the given image; and region-based algorithms, when both pixel values and the surrounding information are utilised to form different regions. Image segmentation is an essential step towards high-level image processing task, such as image analysis, pattern recognition [2,3,4], and computer vision [5]. In different applications of colour image processing, great importance is attached to the techniques used for image segmentation, because the results of the further steps of image processing depend on the segmentation quality (the object recognition and tracking, the retrieval in image databases, etc.).

Numerous image segmentation algorithms have been developed in the literature, from the earliest methods, such as thresholding [6], region growing [7,8], *k*–means clustering [9], watersheds [10], to  more advanced methods, such as power watershed [11,12,13], watershed-cut [14], mutex watershed [15], active contours [16,17], graph cuts [18,19,20,21,22], Markov random fields [23], and sparsity based methods [24]. The interested reader may refer to ([25] Section 2) for an exhaustive review of the literature regarding segmentation algorithms. Moreover, segmentation techniques can be further classified into several classes. In particular, it is possible to consider semi-automatic and automatic algorithms. The semi-automatic approach requires user intervention. A common scenario has the user marking each of the objects of interest, with each mark corresponding to a given object and indicating a small number of pixels that are contained within that object. Other types of user inputs, such as bounding boxes and the like, are possible as well. In any case, the user input should be simple enough to be given in a short time. Semi-automatic segmentation is an attractive approach both for applications (e.g., in biomedical imaging [26]), and from the algorithmic perspective. For example, a large scientific interest lies in how the information spreads from a small set of known samples (the user input) to the entire image: in [27] a Susceptible–Infectious–Recovered (SIR) model is applied to image segmentation task.

At first, the segmentation techniques were mainly proposed for grey-level: the reason is that processing colour images requires computational times considerably larger than those needed for grey-level images, although colour information permits a more complete representation of images and more reliable segmentations. It has long been recognised that the human eye can differentiate thousands of colour shades and intensities but only two dozen shades of grey. For some class of segmentation problems, using grey-scale only does not provide reliable result, for example due to the low contrast or to similar intensity values of different objects. As compared to monochrome images provide further information in addition to simple intensity levels. Colour image processing has thus become increasingly more attractive, although most of the techniques for colour images are derived from monochrome image segmentation. The techniques for segmentation of monochrome images are based on the several principles, such as histogram thresholding, edge detection, and region growing. These principles are employed in many colour image segmentation algorithms, together with different colour models (e.g., RGB, L${}^{*}$a${}^{*}$b${}^{*}$, HSV). To reduce the gap between the computed segmentation and the one expected by the user, these properties tend to embed the perceived complexity of the regions and sometimes their spatial relationship as well [28].

One of the main assumptions in colour image segmentation framework is that homogeneous colours in the image correspond to separate clusters, and, hence, meaningful objects in the image. In other words, each cluster defines a class of pixels that share similar colour properties. As the segmentation results depend on the used colour space, there is no single colour space that can provide acceptable results for all kinds of images. For this reason, many authors tried to determine the colour space that will suit their specific colour image segmentation problem [29,30]. If we consider the image as a graph whose vertices are the image pixels, similarity between pixels in terms of colour or texture features is modelled by a weight function defined on the set of vertices. The weights can be calculated based on appropriate distance functions defined in a suitable colour space [31]. In several papers (see, e.g., [30,32,33,34,35]) the segmentation problem was rephrased in this graph framework by means of the graph cut objective function. Follow-up works on the use of graph based approaches are, for instance, ref. [36] where an iterative application of heat diffusion and thresholding, also known as the Merriman–Bence–Osher (MBO) method is discussed for binary image labelling, and [37] where the Mumford–Shah model is reinterpreted in a graph setting. We point out that most of these methods rely on non-quadratic energies, thus demanding the use of sophisticated and computationally costly optimisation tools [38,39,40]. Ensuring accuracy and smooth solution is also an issue for existing methods. Eventually, we mention machine learning approaches [41,42,43] which reveal to be really powerful in the case one possess a huge image dataset for the training phase.

In this work we present a novel local/global method for semi-automatic multi-label segmentation. The main innovations introduced are:

- The development of a similarity index/distance between pixel using a given colour space and involving pixels in a neighbourhood, in order to improve the random walker approach and a basic clustering step;
- A modified energy related to the random walker approach which improves the quality of the image segmentation and considers only the minimisation of a quadratic function;
- A combination of the above techniques, which overcomes the issues presented by those approaches when they are applied alone;
- A machine-learning approach to adapt the weights of the colour distance (modifying hence the Euclidean distance), acting as a preprocessing on the images.

The interest for a modified energy related to the random walker has been considered by others Authors, see, e.g., [25,27], by using some kind of suitable coordinates or some post-processing step of the probabilities map obtained with random walk approach. We point out that our method involves in a different non-linear way two terms and it is not a thresholding post-process step. In fact, the colour distances affect at the same time the similarity between labelled and unlabelled pixels and on the construction of the graph for the part of the random walk. Due to the connection between the random walk method and the discrete Dirichlet problem, we could consider it as a Laplacian-based manifold method with application to more general data and with some theoretical justification [44].

The proposed method is applied and tested using some benchmark images, together with a series of numerical tests on different colour spaces, a comparison with the *k*–means algorithm and the original random walker method [45] to assess the robustness of the proposed procedure with respect the presence of noise. Two public datasets are used for a performance comparison with state-of-the-art algorithms, namely the normalised random walk and the normalised lazy random walk [27]. The paper further presents a discussion about the properties and the possible developments of the approach.

The remaining sections of the paper are organised as follows. Section 2 introduces the new method and the random walker segmentation algorithm. Moreover, we will also discuss a new definition of non-local distance between pixels. Section 3 is devoted to the numerical experiments. In this section we address the problem of learning suitable weights for the novel colour distance discussed in Section 2. Following the findings of the case study, the conclusions is presented in the last Section 4.

## 2. An Improved Image Segmentation Method

The problem of semi-automatic or interactive segmentation has attracted quite a bit of interest from the computer vision, image processing, and computer graphics communities over the last years. The general idea is to segment an image into two or more separate regions, each corresponding to a particular object (or the background), with the aid of some user input. The goal of this work is to segment images into homogeneous colour-texture regions. The proposed approach does not attempt to estimate a specific model for a texture region, instead it tests for the homogeneity of a given colour-texture pattern in a region. In order to identify this homogeneity, the following assumptions about the image are made:

(a) The image contains a set of approximately homogeneous colour regions (avoid segmentation too granular or too noisy);
(b) The colour information in each image region can be represented by a set of few quantised colours (we can consider some kind of colour categorisation model);
(c) The colours between two neighbouring regions are distinguishable (a suitable definition for similarity between pixels).

**Figure 1:**
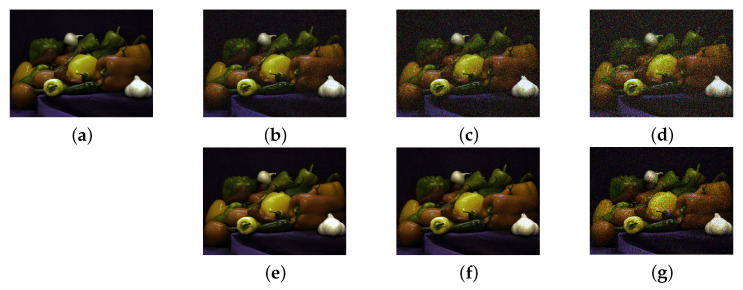
Example of several noise levels of different types. (**a**) Classical peppers image from the default image dataset of MatLab. (**b**–**d**) peppers image affected by Gaussian noise of level 0.1, 0.4, and 0.7, respectively. (**e**–**g**) pepper image with different Poisson noise levels. The Poisson noise has been added via the MatLab function imnoise.

The first assumption requires images that present several regions with similar colour. Moreover, in practical application (such as in astronomical or medical imaging) the images are perturbed by noise, which is due to the physics beyond image acquisition process (see [46] for a deeper insight on the topic): this requirement asks for a noise level that allows to distinguish the different coloured regions. Figure 1 presents several cases of different noise levels (see Section 2.3 Equation (18) for the Gaussian case).

Recently, a growing interest is attracted by an interactive graph based image segmentation algorithms such as graph cut [47] and random walker (RW) [25,27,45] algorithms. The random walker algorithm represents a recent noteworthy development in the weighted graph-based interactive segmentation methods. This technique with user interaction is more suitable for volumetric medical images to guarantee the reliability, accuracy, and fast speed demands.

### 2.1. The Random Walker Method

The framework of the RW involves an undirected graph G=(V,E), where *V* and *E* are the set of vertexes and the set of edges, respectively. The set of the vertex $V=\{v_{i}\}$ is the set of pixels present in the image, whose number is denoted with |V|. The vertex set can be partitioned into two further sets: $V=V_{m}⋃V_{u}$. One set is “marked vertices” $V_{m}$, which are marked by user, also called seeds, as belonging to the several objects, and the rest of the image pixels is the set $V_{u}$ or the “unlabelled vertices”. The set of the edges *E* consists of the pairs of pixels which are neighbours in the image, e.g., standard 4-neighbourhood or 8-neighbourhood. We denote with $e_{ij}$ the edge linking the vertexes $v_{i}$ and $v_{j}$.

The weight of an edge $e_{ij}$ can be represented by a function $\omega (v_{i},v_{j})$ based on the intensities’ difference of the two pixels.

For example, suppose we consider the classical RGB colour coordinates of a pixel corresponding to a vertex $v_{i}$: this consists in a vector $C\left(v_{i}\right)\in R^{3}$. Denote with $d\left(v_{i},v_{j}\right)=∥C\left(v_{i}\right)-C\left(v_{j}\right)∥$ a colour distance between two pixels, namely $v_{i},\;v_{j}$, being ∥·∥ a norm in $R^{3}$. A classical choice for the RGB space is

(1) $$d\left(v_{i},v_{j}\right)=\sqrt{{\left(r_{i}-r_{j}\right)}^{2}+{\left(g_{i}-g_{j}\right)}^{2}+{\left(b_{i}-b_{j}\right)}^{2}}$$

where ${\left(r_{i},g_{i},b_{i}\right)}^{⊤}$ are the colour coordinates of the pixel $v_{i}$. Using a different colour spaces may induce to employ more suitable other distances. For example, in the CIE L${}^{*}$a${}^{*}$b${}^{*}$ space the following distances [48] can be used

(2) $$\begin{array}{cc} d(v_{i},v_{j}) & =\Delta E_{ab}^{*}\left(v_{i},v_{j}\right)=\sqrt{{\left(L_{i}^{*}-L_{j}^{*}\right)}^{2}+{\left(a_{i}^{*}-a_{j}^{*}\right)}^{2}+{\left(b_{i}^{*}-b_{j}^{*}\right)}^{2}} \end{array}$$

(3) $$\begin{array}{cc} d(v_{i},v_{j}) & =\Delta E_{94}^{*}\left(v_{i},v_{j}\right)=\sqrt{{\left(\frac{L_{i}^{*}-L_{j}^{*}}{k_{L}S_{L}}\right)}^{2}+{\left(\frac{\Delta C_{ab}^{*}}{k_{C}S_{C}}\right)}^{2}+{\left(\frac{\Delta H_{ab}^{*}}{k_{H}S_{H}}\right)}^{2}} \end{array}$$

where

$$\begin{array}{cc} C_{ab}^{*} & =\sqrt{{a_{i}^{*}}^{2}+{b_{i}^{*}}^{2}}-\sqrt{{a_{j}^{*}}^{2}+{b_{j}^{*}}^{2}}\\ \Delta H_{ab}^{*} & =\sqrt{{\left(a_{i}^{*}-a_{j}^{*}\right)}^{2}+{\left(b_{i}^{*}-b_{j}^{*}\right)}^{2}-{\Delta C_{ab}^{*}}^{2}}\\ S_{L} & =1,\;S_{C}=1+K_{1}\sqrt{{a_{i}^{*}}^{2}+{b_{i}^{*}}^{2}}\\ S_{H} & =1+K_{2}\sqrt{{a_{i}^{*}}^{2}+{b_{i}^{*}}^{2}} \end{array}$$

with $k_{C}=k_{H}=1$ and $k_{L},K_{1},K_{2}$ depend on the application.

Once the couple colourspace/colour distance is chosen, some possible choices for weights are

(4) $$\omega \left(v_{i},v_{j}\right)=e^{-\beta \;d{\left(v_{i},v_{j}\right)}^{2}}\;\mathrm{or}\;\omega \left(v_{i},v_{j}\right)=\frac{1}{\varepsilon +\sigma \;d(v_{i},v_{j})}$$

where the value of the parameters β,σ,ε>0 can be tuned accordingly. The weights of the edge lie in the range (0,1), for similar pixels we will have a weight close to 1, whereas for very different pixels the weight is close to 0. Having the above graph structure in hand, the idea of the RW method is as follows. It is assumed that image consists of *K* possible regions (objects) and each labelled vertices of $V_{M}$ belongs to one of these *K* regions. If we consider a weighted edge $e_{ij}$ whose endpoints are $v_{i}$ and $v_{j}$, the weight of the edge $\omega \left(v_{i},v_{j}\right)\in \left(0,1\right)$ can be interpreted as the measurement of transition probability of a random walk from one vertex to another vertex. Depending on the weight of the edge, the random walk is likely to transition form $v_{i}$ to $v_{j}$ if the vertexes are very similar in colour, and is unlikely to move from $v_{i}$ to $v_{j}$ if they are very dissimilar. Given the above probabilities, the segmentation algorithm computes the probability for each vertex $v_{i}$ that a random walker leaving that pixel reaches any one of the labelled vertices belonging to the *k*-th object: we denote this probability by $x_{i}^{k}$. Then the image segmentation is done according to these probabilities. More specifically, for any vertex $v_{i}$, we classify it as belonging to the *k*-th region if $x_{i}^{k}>x_{i}^{\bar{k}}$ for all $\bar{k}\neq k$. We observe that edges in the image correspond to low transition probabilities, as they involve a rapid change in colour or intensity. Thus, this algorithm will tend to respect image edges in performing the segmentation.

It was shown in [45] that these probabilities may be calculated analytically by solving a linear sparse system of equations with the graph Laplacian matrix. The Laplacian matrix is defined as

(5) $$L_{ij}=\left\{\begin{array}{cc} d_{i}, & \mathrm{if}\;i=j\\ -\omega (v_{i},v_{j}), & \mathrm{if}\;v_{i}\;\mathrm{and}\;v_{j}\;\mathrm{are}\;\mathrm{adjacent}\;\mathrm{nodes}\\ 0, & \mathrm{otherwise}, \end{array}\right.$$

where $L_{ij}$ is indexed by vertices $v_{i}$ and $v_{j}$, and 

$$d_{i}=\sum \omega \left(v_{i},v_{j}\right)$$

for all edges $e_{ij}$ incident on vertex $v_{i}$. Assuming that each node $v_{j}\in V_{m}$ has also been assigned with a label *k*, we can compute the probabilities, $x_{k}={\left(x_{1}^{k},x_{2}^{k},\cdots ,x_{\left|V\right|}^{k}\right)}^{⊤}$, that a random walker leaving node $v_{i}$ arrives at a marked node $v_{j}$ by solving the minimisation of

(6) $$E\left(x_{k}\right)\;=\;\frac{1}{2}\sum_{(v_{i},v_{j})\in E}\;\omega \left(v_{i},v_{j}\right){\left(x_{i}^{k}-x_{j}^{k}\right)}^{2}\;=\;\frac{1}{2}x_{k}^{T}\;L\;x_{k}.$$

Since L is positive semi-definite, the only critical points of *E* will be minima. Note also that the solution x that minimises *E* is also called combinatorial harmonic function [49], because the corresponding continuous problem leads to the minimisation of the Dirichlet integral via harmonic functions. Moreover, the problem

(7) $$x_{D}=\underset{\begin{array}{c} x \end{array}}{\mathrm{argmin}}\;\;E\left(x\right),$$

is also called combinatorial Dirichlet problem.

We consider the partition of the vertices into two sets, namely $V_{m}$, the marked vertices by the user, and $V_{u}$, the unmarked nodes, such that $V_{m}⋃V_{u}=V$ and $V_{m}⋂V_{u}=\emptyset$. Note that $V_{m}$ contains all marked points, regardless of their label. We may assume without loss of generality that the nodes in L and x are ordered, such that marked nodes are first and unmarked nodes are second. Therefore, we may decompose (with abuse of notation) the above formula into

(8) $$E\left(x_{m},x_{u}\right)=\frac{1}{2}\left[x_{m}^{⊤},\;x_{u}^{⊤}\right]\left[\begin{array}{cc} L_{m} & B\\ B^{⊤} & L_{u} \end{array}\right]\left[\begin{array}{c} x_{m}\\ x_{u} \end{array}\right],$$

where $x_{m}$ and $x_{u}$ correspond to the probabilities of the marked and unmarked nodes, respectively, while B represents the anti-diagonal blocks of the Laplacian. Moreover, for simplicity of notation we omit here the index *k*. The same problem could be interpreted as an interpolation of missing data: indeed, assume that we have a graph where we have defined some (numerical) values for a subset of the vertices (our labelled nodes), and that we want to somehow fill in the missing data for the remaining nodes.

The Equation (8) reads,

(9) $$E\left(x_{m},x_{u}\right)=\frac{1}{2}x_{m}^{⊤}L_{m}x_{m}+x_{u}^{⊤}B^{⊤}x_{m}+\frac{1}{2}x_{u}^{T}L_{u}x_{u}$$

and the unknowns are the entries of the vector $x_{u}$. Differentiating *E* with respect to $x_{u}$ and finding the critical point, yields

(10) $$L_{u}x_{u}=-B^{⊤}x_{m}$$

which is a system of linear equations with $|V_{u}|$ unknowns. If the graph is connected, or if every connected component contains a seed, then this equation will be non-singular. Define the set of labels for the marked vertices as a function $Q(v_{j})=k$, $\forall v_{j}\in V_{M}$, where k∈N, 0<k≤K. Let $m^{k}\in R^{|V_{m}|}$ for each label *k*, at vertex $v_{j}\in V_{m}$ as

$$m_{j}^{k}=\left\{\begin{array}{cc} 1, & \mathrm{if}\;Q(v_{j})=k\\ 0, & \mathrm{if}\;Q(v_{j})\neq k. \end{array}\right.$$

Therefore, for label *k*, the solution to the combinatorial Dirichlet problem (7) may be found by solving

(11) $$L_{u}x_{u}^{k}=-B^{⊤}m^{k}.$$

Thus, each unlabelled pixel gets *K* probabilities which indicate that a random walker starting from the unmarked pixel reaches each *k*-marked region. Eventually, the label assigned to each unlabelled pixel corresponds to the index in the solution of (10) of the largest probability. For example, suppose that an image contains only K=3 marked regions. For sake of simplicity, consider just one pixel $\tilde{x}$: the solution of (10) for this pixel reads as ${\tilde{x}}_{u}=\left({\tilde{x}}^{1},{\tilde{x}}^{2},{\tilde{x}}^{3}\right)={\left(0.1,0.6,0.3\right)}^{⊤}$: this means that a random walker starting from such a pixel has a probability of reaching the region k=1 equal to 0.1, it has a probability of reaching the region corresponding to the label k=2 of 0.6 and eventually with probability of 0.3 it reaches the region marked as k=3. Then, this unmarked pixel shall be labelled with k=2 since a random walker is more likely attracted from the region k=2. This approach is adopted also in [25].

### 2.2. A Suitable Similarity Measure

In many real-world applications, the object of interest may contain several colour shades: thus, the RW approach may encounter some issues in recognising pixels belonging to the same object, due the large influence of the colour distance in the Laplacian formulation. This imposes an unusual constraint on the RW algorithm if the weight between two pixels is solely based on the Euclidean distance between their respective colour vectors, or even when a more sophisticated measure such as (3) is employed. Moreover, we do possess prior information: the user input in the form of seeds gives us some important information about the colour distribution of the various objects. This further information is exploited in formulating more meaningful edge weights: in particular, we consider not only the colour of a single pixel but also the colours of its adjacent pixels, chosen in a suitable neighbourhood.

**Figure 2:**
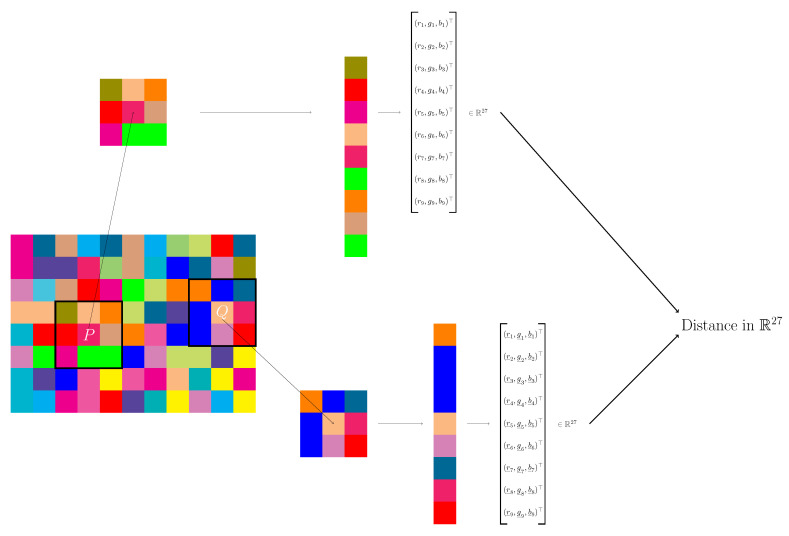
The definition of a new distance between pixel *P* and pixel *Q* in a colour image. We select a neighbourhood for each pixel, here a 8-neighbourhood, and consider the components of each pixel in the neighbourhood in some fixed colour space, in this example the RGB space. For the pixel P we have the vectors $(r_{i},g_{i},b_{i}),\;i=1,...,9$, while for the pixel *Q* the vectors $({\underline{r}}_{i},{\underline{g}}_{i},{\underline{b}}_{i}),\;i=1,...,9$. Finally, we collect all the entries and form two vectors $V_{P}$, $V_{Q}$, and compute some distance, e.g., the Euclidean one, between these two vectors.

We fix a system of neighbourhoods with *N* pixels, for each pixel, for example 8–neighbourhoods, and a colour space: hereafter, we consider, by way of example, the RGB system. Then, for a pixel *P* we consider the vectors $(r_{i},g_{i},b_{i}),\;i=1,...,N$ of the RGB components of the colour for each pixel in the neighbourhood (see Figure 2). Finally, we collect all the entries of the colour vectors in a single vector $V_{P}\in R^{3N}$. We fix a distance $d_{3N}$ in the space $R^{3N}$ and for a couple of pixels *P* and *Q* we compute $d_{3N}\left(P,Q\right)$.

We define hence the similarity index $S\left(P,Q\right)={\left(d_{3N}\left(P,Q\right)\right)}^{-1}$ to be used as weight in (4).

**Remark** **1.** *The proposed similarity index can be seen as the first step of a k-means algorithm, where the starting centroids are computed using as seed the marked regions.*

**Figure 3:**
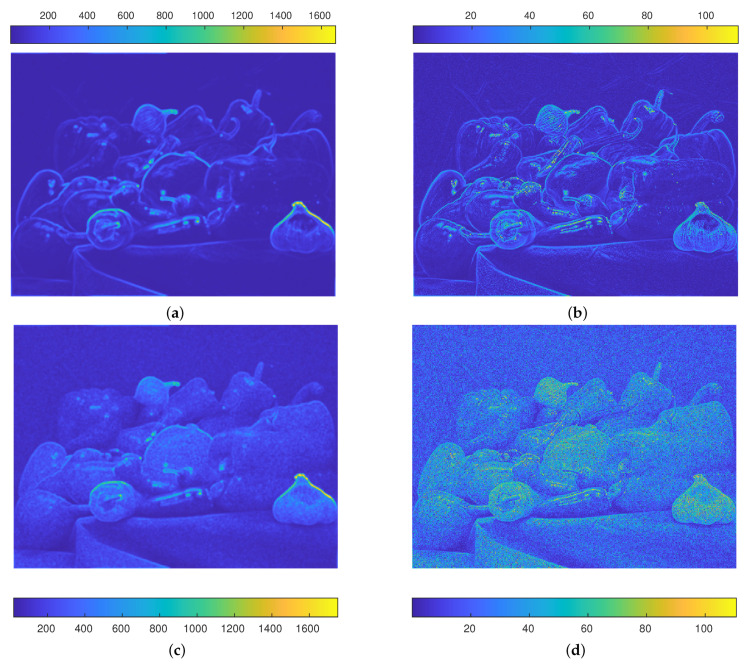
Colour distances on Peppers image. (**a**) proposed similarity index. Each pixel of this image depicts the proposed distance from its 4-neighbours. The smoothing effect promotes the difference between different objects and at the same time uniform coloured regions are discarded. (**b**) each pixel of this image depicts the classical Euclidean distance from its 4 neighbours. Several unwanted details are maintained inside the object of interest. (**c**) proposed similarity index in presence of Poisson noise. This distance preserves the boundary between objects of different colours. (**d**) classical Euclidean distance in presence of Poisson noise. The majority of colour information is lost due to the noise affecting the image.

This similarity, or the distance, could be used in a clustering algorithm and this represents a global measure or comparison between pixels. This distance allows to see the colour of the pixel not as a single information, but in relation to its neighbours. Moreover, using patches instead of single pixels induces a smoothing effect [50] which may provide some advantage in presence of noise: Figure 3a refers to this similarity index applied to the Peppers image, where each pixel contains the value of the distance from the very pixel from its 4 neighbours: one can note that the boundaries between different objects are well emphasised, while the uniform regions inside them have very small values. For example, this means that the distance among the pixels of the yellow pepper at the centre of the image is small, while the distance between the pixels of its boundary and those of the surrounding green and red peppers is large. Figure 3b on the other hand shows that classical Euclidean distance is able to recognise the boundaries too, but at the same time it maintains an high level of details inside the objects and low values. The interest of having high values for these colour distance finds its meaning in Equation (4): the greater the distance the smaller the weight, hence the probability for the random walker to move among objects with different colours is small. Figure 3c shows that the usage of patches is important in presence of noise: the induced smoothing effect controls the influence of the noise, avoiding thus the loss of information as it happens in Figure 3d, where patches were not being considered. Moreover, the influence of the noise can be further reduced by employing larger patches. One may observe that the presence of noise affects also the values of the proposed distance. Indeed, consider a pixel *P*: denote with $V_{P}^{*}\in R^{3N}$ its corresponding colour vector and with $V_{P}∼V_{P}^{*}+N\left(0,\sigma^{2}I_{d}\right)$ its noisy version when Gaussian noise with zeros mean and covariance matrix $\sigma^{2}I_{d}$ is considered, being $I_{d}$ the identity matrix. Consider another pixel *Q*, such that the intersection of the neighbourhoods of *P* and *Q* are empty. We can give an estimation of the expected value $E\left[d_{3N}{\left(Q,P\right)}^{2}\right]$ of the distance

(12) $$E\left[d_{3N}{\left(Q,P\right)}^{2}\right]={∥V_{P}^{*}-V_{Q}^{*}∥}^{2}+2\times 27\sigma^{2}=d_{3N}^{*}{\left(Q,P\right)}^{2}+54\sigma^{2}$$

A similar estimation can be given when the noise affecting the pixels is not addictive but signal dependent: for the case of Poisson noise $V_{P}∼\mathrm{Poiss}\left(V_{P}^{*}\right)$ one obtains

(13) $$E\left[d_{3N}{\left(Q,P\right)}^{2}\right]=∥V_{P}^{*}-V_{Q}^{*}{∥}^{2}+|V_{P}^{*}+V_{Q}^{*}{|}_{1}=d_{3N}^{*}{\left(Q,P\right)}^{2}+{∥V_{P}^{*}+V_{Q}^{*}∥}_{1}$$

where ${∥\cdot ∥}_{1}$ is the $\ell_{1}$ norm in $R^{3N}$ and $d_{3N}^{*}\left(Q,P\right)$ is the similarity index between the clean pixels. The above estimations are based on the fact that for a random variable *X* one has $E\left[X^{2}\right]=E{\left[X\right]}^{2}+\sigma^{2}\left(X\right)$, with $\sigma^{2}\left(X\right)$ the variance of *X*.

This non-local method is inspired by recent approaches in signal analysis [3,51,52].

Now, we consider the vertex labelling function, for simplicity we will consider labels represented by integers,

(14) $$F_{L}:\;V\to S_{L}=\left\{1,\;2,\ldots ,\;K\right\},\;\;\;K\in N,\;K>1$$

which associates a label in a certain set to each vertex (pixel). We combine the RW approach, with the new distance defined above, and the new similarity measure and define $F_{L}$ as

(15) $$F_{L}\left(v_{i}\right)=\underset{\begin{array}{c} k\in S_{L} \end{array}}{\mathrm{argmax}}\;\;\;\left(S{\left(i,k\right)}^{\alpha }\;{\left(x_{i}^{k}\right)}^{\beta }\right)$$

where α≥0, β≥0 are two parameters introduced for adding flexibility to the algorithm and to provide different weights to the two components of the labelling function. Due to the concavity of the logarithm function, and the positivity of S(i,k), and $x_{i}^{k}$, we can rewrite the labelling problem in an equivalent way as follows

(16) $$F_{L}\left(v_{i}\right)=\underset{\begin{array}{c} k\in S_{L} \end{array}}{\mathrm{argmax}}\;\;\log\left(S{\left(i,k\right)}^{\alpha }\;{\left(x_{i}^{k}\right)}^{\beta }\right)=\underset{\begin{array}{c} k\in S_{L} \end{array}}{\mathrm{argmax}}\;\;\left(\alpha \log\left(S\left(i,k\right)\right)+\beta \log\left(x_{i}^{k}\right)\right).$$

The proposed method, therefore, can be summarised in Algorithm 1.

**Remark** **2.** *The two terms in the functional in (16) could be considered as a “fidelity term”, the αlog(S) part, and a regularising term, the βlog(x) part.*

**Algorithm 1** Random walk by colour similarity algorithm (RaWaCS)
Set the parameters α, β, the neighbours system and the similarity function.Acquisition of user-marked pixels.Compute the global similarity index S(i,k) for any $v_{i}\in V_{u}$ and $v_{k}\in V_{m}$.Solve systems (10) for any labelled vertices in $V_{m}$, where the Laplacian matrix uses the index computed at step 3.Evaluate the labelling function as in (16).



In the next section we will discuss the proposed combination of RW probabilities and similarity index. The weights in Equation (6) are chosen as

(17) $$\omega \left(v_{i},v_{j}\right)=\frac{1}{S(i,j)+\varepsilon }\;,\;\varepsilon =10^{-3}$$

### 2.3. Combined Role of the Similarity Index and Random Walk Approach

**Figure 4:**
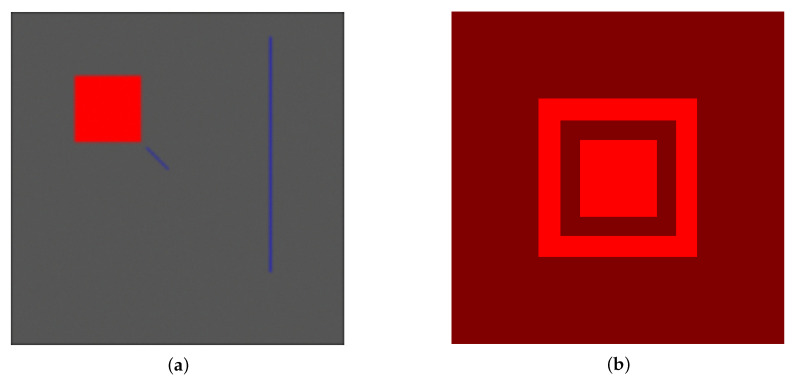
(**a**) a simple RGB image with a red square and a couple of blue lines. The red coordinates are ${\left(1,0,0\right)}^{⊤}$ in the RGB space, while the lines’s coordinates are ${\left(0,0,1\right)}^{⊤}$. The background value is constant and set to 1/3 on all channels. A 7×7 Gaussian PSF with unitary variance blurs the whole image, and Gaussian noise at level 0.1 is added to each channel. (**b**) a red square surrounded by frames; one of the frame has the same colour coordinates for the inner square, the other two frames share the same colour.

We consider two different images to justify the introduction of the similarity index S and its non-linear combination in (15), showing that the usage of one of the two techniques alone is less performant than the combined approach. The first test image is a simple one, depicted in Figure 4: the background is set to grey at level 0.33, while each pixel of the square is set to red ${\left(1,0,0\right)}^{⊤}$ in RGB coordinates. The pixel of the blue lines are set to ${\left(0,0,1\right)}^{⊤}$. The image is blurred with a 7×7 Gaussian Point Spread Function (PSF) of zero mean and unitary variance; each channel of the image is affected by Gaussian noise, using the formula

(18) $$G_{n}\left(:,:,i\right)=G\left(:,:,i\right)+\sigma_{n}\frac{\eta_{i}}{∥\eta_{i}{∥}_{F}}{∥G\left(:,:,i\right)∥}_{F},\;i=1,2,3$$

where η is the realisation of a multivalued random Gaussian variable of zero mean and unitary variance; $\sigma_{n}$ is the noise level and it is set to 0.1; ${∥\cdot ∥}_{F}$ is the Frobenius norm, see [53] for technical details. The second image consists in a red square at the centre, surrounded by three frames: the colour coordinates of the red square are ${\left(1,0,0\right)}^{⊤}$, while the colour coordinates of the frames are ${\left(0.5,0,0\right)}^{⊤},\;{\left(1,0,0\right)}^{⊤},\;{\left(0.5,0,0\right)}^{⊤}$, respectively.

**Figure 5:**
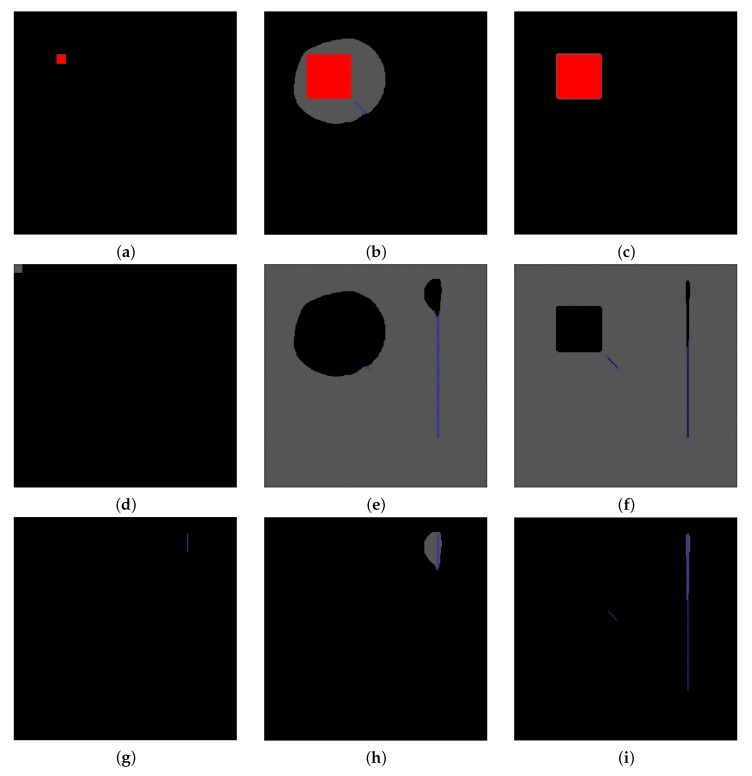
Comparison between simple labelling using the diffusion process and the proposed procedure. (**a**,**d**,**g**) refer to marked red, blue, and background regions, respectively. (**b**,**e**,**h**) refer to the labelled regions by employing only the diffusion process (α=0,β=1). (**c**,**f**,**i**) show the results of the proposed procedure (α=β=1) for each region.

The first test shows that the sole diffusion process provides unreliable results on the image of Figure 4a. The first column in Figure 5 depicts the marked regions for the labelling process. The results in the second column of Figure 5 show that the diffusion process, due to its local behaviour and to the influence of the noise, overestimates the red region whilst the blue line is recognised only in its upper part together with a large part of the background. Once the similarity comes into play, as shown in the 3rd column of the same figure, the labelling process is correct, the influence of the blurring effect of the PSF and of the noise is under control. Moreover, the introduction of the similarity index allows to recognise also the diagonal line close to the red square: this type of lines are hardly individuated by the diffusion process due the construction of the Laplacian, which is based on the 4 nearest neighbours.

**Figure 6:**
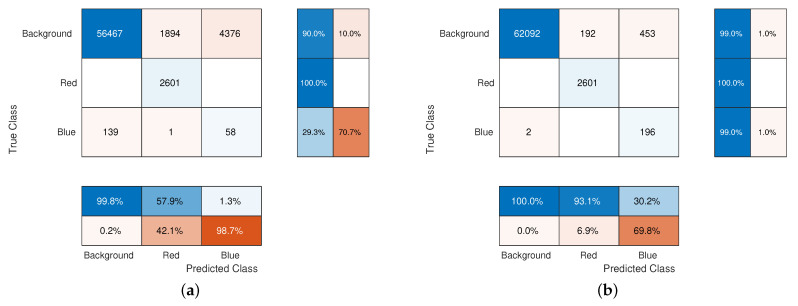
(**a**) confusion matrix with α=0. (**b**) confusion matrix with α=1, respectively. The influence of the similarity index is evident: its presence allows to decrease the percentage of background pixels classified as red and blue pixels from to 42.1% to 6.9% and from to 98.7% to 69.8%, respectively, while at the same time the performance of correctly labelled blue pixels increases from 29.3% to 99.0% (the success rate for the red ones is 100% in both cases).

In order to numerically evaluate the difference in performance, we compute the confusion matrix of the labelling process: the (i,j)-th element of this matrix provides the number of pixels belonging to the *i*-th class which are recognised as elements of the *j*-th class. The diagonal contains the total of correctly labelled pixels. Figure 6 shows the confusion matrices related to the two experiment with α=0 and with α=1. In the former case, even if the red square is fully recognised a large area of the background is included in this class: the 42.1% of the pixels labelled as “red” are actually belonging to the background, whilst with α=1 this percentage falls to 6.9%. Furthermore, the blue lines are poorly recognised in the first case: indeed only the 29.3% of the pixels are recognised and more than 70% of the pixels labelled as “blue” are actually background pixels. As soon as we introduce the similarity index, the blue lines are recognised, even if in this case too some pixels of the background are included in this class. Eventually, the similarity index induces a small increment in the performance for recognising the background pixels. The RaWaCs algorithm overcomes a simple random walk approach, even if the latter employs a suitable colour distance.

**Figure 7:**
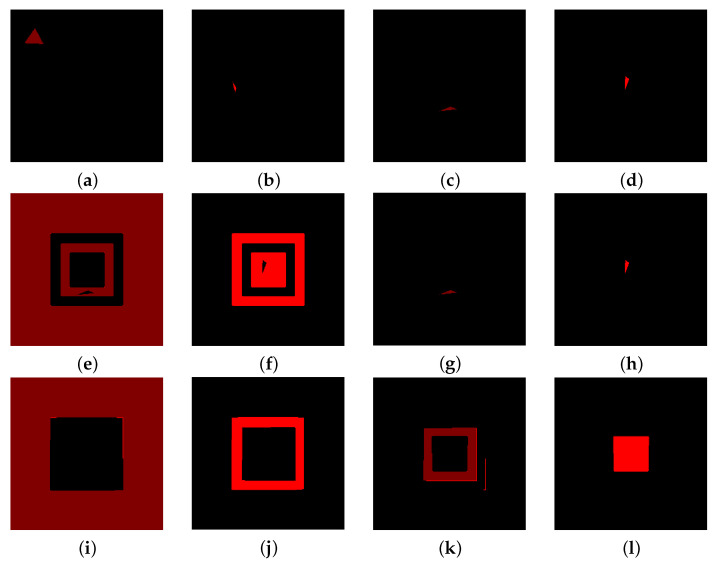
(**a**–**d**) marked regions for the labelling of the four different objects. (**e**–**h**) results provided by the employment of the sole similarity index. (**i**–**l**) results of the proposed procedure. In the last case, the objects are fully recognised.

The second test is performed on the image in Figure 4b: if the interest lies in recognising all the objects in the image, the similarity index may fail in this task when several objects share the same exact colour. Indeed, marking the 4 different region in Figure 4a, namely the centred square and the three frames (see Figure 7, first column), and using only the index S to label them provide with poor results: the regions with the same colour are completely recognised, even if they belongs to different objects. For example, the two frames with colour coordinates ${\left(0.5,0,0\right)}^{⊤}$ are labelled as they both belongs to the object marked in Figure 7a, whilst the inner one actually belongs to the region marked in Figure 7g. Using the RaWaCs algorithm with β≠0, on the other hand, let us achieve reliable results (see Figure 7, last column): the diffusion indeed mediates the influence on the final result of the similarity index, denying the propagation of the labelling process to regions of the same colour but originally marked with a different label.

### 2.4. Peppers

**Figure 8:**
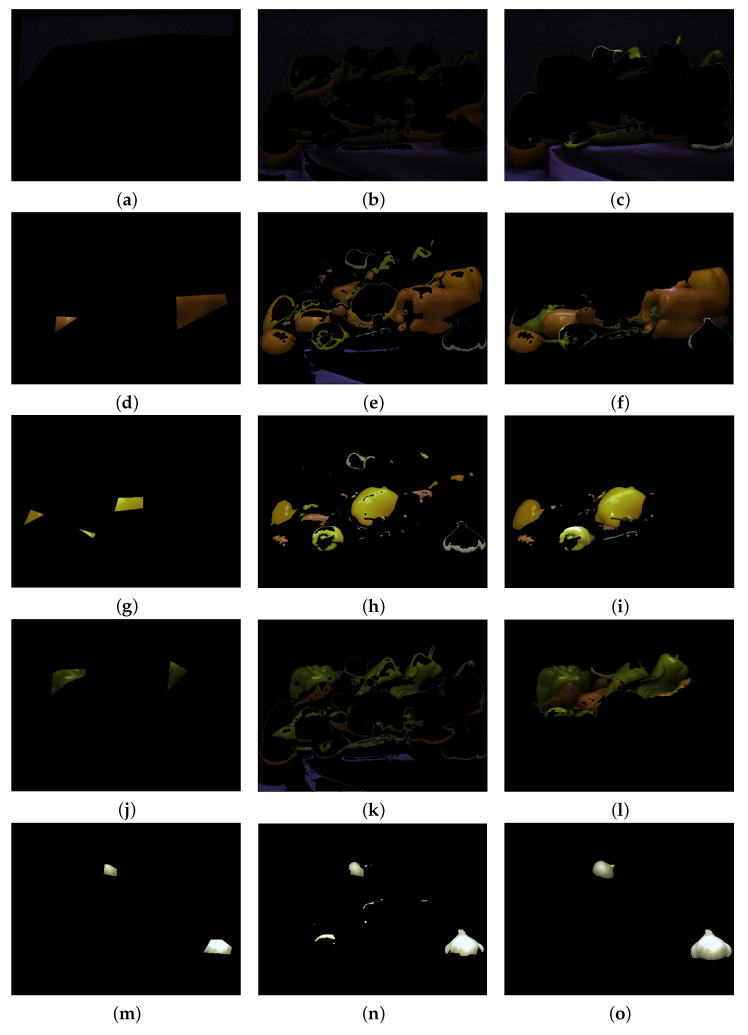
(**a**,**d**,**g**,**j**,**m**) marked regions, which refers to background, red, yellow, green peppers, and ail and onion, respectively. (**b**,**e**,**h**,**k**,**n**) labelled region obtained by employing the sole similarity index. (**c**,**f**,**i**,**l**,**o**) results of the proposed procedure with α=β=1.

A further experiment carried on the classical pepper image shows that the simple similarity index fails in recognising different objects with similar colours. This task is more challenging than separating a single object from the background. In this experiment the aim consists in separating vegetables with the same colour in different classes and to distinguish them from the background. We have then 5 marked regions: background, red peppers, yellow peppers, green peppers, ail and onion. The second column of Figure 8 refers to the result obtained via only S, which means that we considered only the distance between each pixel of the image and the centroids of the marked region. This leads to unsatisfactory results: indeed, this index forces the second label (namely, the “red pepper” one) to include some parts of the violet blanket and some spots of green peppers, while the third label (“yellow pepper” one) embraces also some regions belonging to red peppers and to ail and onion. The fifth label, i.e., the label that enclose the white parts of the image, includes also some bright spots that belong to yellow regions of the image. Once Algorithm 1 with α=β=1 is employed, the objects in the image are very well recognised: the background now includes the entirety of the violet blanket, with some boundary parts of the vegetables, the red regions include a lesser amount of green parts. The best results are achieved in the case of the 3rd and 5th label, the yellow and the white regions, while the green peppers still include some red ones. A visual inspection shows anyway that the proposed formula yields better results than the mere application of the similarity index.

## 3. Results

This section is devoted to show the performance of Algorithm 1. The first set of experiments shows that the RaWaCs procedure is really robust with respect to the noise level perturbing the image. The comparison is done with the classical *k*-means algorithm and the random walk method [45], the latter using a classical Euclidean distance between pixels without taking into account the neighbourhoods. A second experiment is carried on a database with 200 biological images, containing cells and their nuclei: since this database includes the ground truth segmentation, we are able to check the performance of the proposed algorithm. In order to prove the robustness of our approach, we apply it also to the GrabCut database, which contains 49 images of different nature: in this case, the main aim is to separate a single object from the background, while the proposed procedure is particularly tailored for the segmentation of object with similar colours. The third set of experiments shows how our algorithm behaves depending on different colourspaces. We address also the learning of the weights to be used in the computation of the colour distance. Eventually, the last section is devoted to asses the quality of the segmentation carried on biological images.

All the experiments were carried on a laptop equipped with Linux 19.04, with an Intel(R) Core(TM) i5–8250U CPU (1.60 GHz), 16 GiB RAM memory (Intel, Santa Clara, CA, USA) and under MatLab R2020b environment (MathWorks, Natick, MA, USA).The code is available at https://github.com/AleBenfe/RaWaCs (accessed on 1 September 2021).

### 3.1. Comparison with *k*-Means and Classical Random Walk in Presence of Additive Noise

When the interest lies in recognising different objects that share the same colour, classical algorithms solely based on single–pixel colour information may fail in this task. This section is devoted to compare the performances of Algorithm 1 with two state-of-the-art algorithms: *k*-means strategy and classical random walker method [45]. The test image in Figure 4a is employed and Gaussian noise is added to the clean image, with different noise levels. For this comparison, we use the same user-marked regions shown in Figure 5a,d,g and we compare the results obtained via the *k*-means algorithm (given by the MatLab function kmeans, set with standard options and maximum number of iteration equal to 1000) and the ones obtained via the random walker method, using the Matlab code available in [54]. Two performance measures are employed to asses the quality of the segmentation process for each label: the normalised volume difference (NVD) and the normalised object overlap (NOO)

(19) $$\mathrm{NVD}=\frac{\sum_{i=1}^{\left|V\right|}\left|g_{i}^{k}-s_{i}^{k}\right|}{\sum_{i=1}^{\left|V\right|}g_{i}^{k}},\;\mathrm{NOO}=\frac{\sum_{i=1}^{\left|V\right|}g_{i}^{k}s_{i}^{k}}{\sum_{i=1}^{\left|V\right|}g_{i}^{k}+s_{i}^{k}-g_{i}^{k}s_{i}^{k}}$$

where $g^{k}$ is the ground truth for the label *k*:

(20) $$g_{i}^{k}=\left\{\begin{array}{cc} 1 & \mathrm{if}\;\mathrm{vertex}\;i\;\mathrm{belongs}\;\mathrm{to}\;\mathrm{label}\;k\\ 0 & \mathrm{otherwise} \end{array}\right.$$

and $s^{k}$ is the result of the segmentation:

(21) $$s_{i}^{k}=\left\{\begin{array}{cc} 1 & \mathrm{if}\;\mathrm{vertex}\;i\;\mathrm{has}\;\mathrm{been}\;\mathrm{labeled}\;\mathrm{with}\;\mathrm{label}\;k\\ 0 & \mathrm{otherwise} \end{array}\right..$$

**Figure 9:**
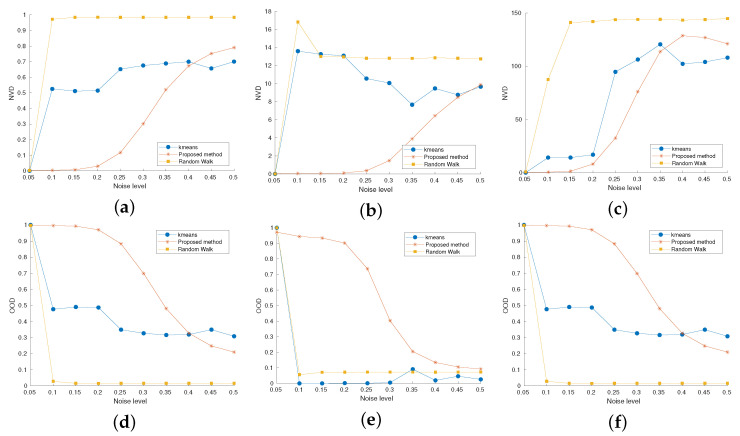
Performance comparison between the RaWaCs algorithm (orange), the *k*-means algorithm (blue) and the random walk method (yellow) with respect to the noise level. (**a**–**c**) NVD for labels referring to the background, the red square and the blue lines of Figure 4a. (**d**–**f**) OOD for labels referring to the background, the red square and the blue lines of Figure 4a. The last label presents high challenges in its segmentation due to its thinness and orientation.

A reliable segmentation technique should provide a low NVD and an high NOO: see [55] for more details about these measures. The plots in Figure 9 depict the behaviour of such measures wrt increasing noise level, when Gaussian noise is added to the image (see Equation (18)). The RaWaCs procedure reveals to be very robust with respect to the noise in both indexes: the random walk method and the *k*-means approaches suffer from the presence of the noise starting from $\sigma_{n}=0.1$.

### 3.2. WBC and GrabCut Datasets

**Figure 10:**
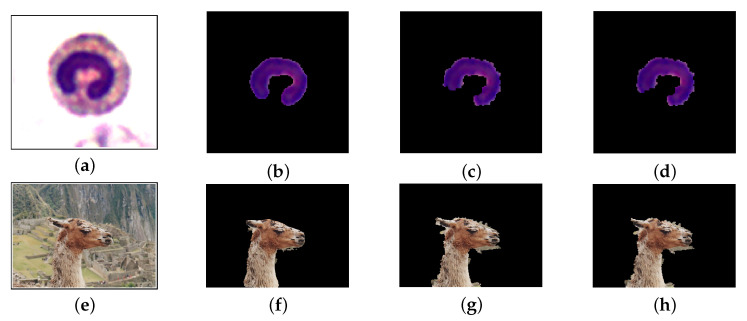
Segmentation results: first row refers to an image of the WBC dataset, while the second row refers to the llama image of the GrabCut dataset. (**a**,**e**) original image. (**b**,**f**) RaWaCs. (**c**,**g**) NRW. (**d**,**h**) NLRW.

We consider 200 images of the white blood cell (WBC) database [56], which contains images of cells with their nuclei. This database contains also the ground truth, where the nuclei, cytoplasm and background (that may contains also blood cells) were marked by domain experts. The dimension of each image is 120×120 pixels and the colour-depth is 24 bit. See [56] for the technical details on the image acquisition procedure. We compare our method with the classic random walk (RW) [45], with the normalised random walker (NRW) and with the normalised lazy random walker (NLRW) [27,57], using three further performance measures: the Rand index (RI), the global consistency error (GCE), and the error rate (ERR). The former measures how the segmentation and the ground truth agree, by counting the pixels marked with the same labels: the higher this score is, the better the performance is. The GCE index measures the refinement level between two segmentations: in this case low values mean good performances. The error rate measures the percentage of misclassified pixels. Eventually, we consider the computational time employed for the segmentation of a single image: we used the tic-toc” MatLab function. For this experiment, we selected the same manually marked regions for all the 4 procedures. Table 1 contains the results of this experiment: it shows that Algorithm 1 has a remarkable performance in comparison to the other method. Regarding the computational time, the classic random walk method is faster, but on the other hand its RI index is slightly lower than the one of the RaWaCs. Figure 10 (first row) provide a visual inspection of the performances of these algorithms. All the results are obtained by setting β=1 and α=2 in Equation (16): these parameter might be suboptimal for some images.

The second dataset employed to assess the performance of the proposed procedure is the GrabCut dataset [19], which contains different images of different dimensions. This dataset was created mainly to test algorithms whose main aim is separating the foreground (e.g., an animal, a car, a person, a vase) from the background. Even if the algorithm presented in this work is not really tailored for this task, Table 2 shows that the performances are remarkable, in comparison with the other 3 algorithm considered for this benchmark. The NLRW performs a little bit better, but it requires a larger amount of computational time. Figure 10 (second row) shows a visual example of the obtained results.

### 3.3. Different Colour Spaces

This subsection is devoted to evaluate the performance of Algorithm 1 on different colourspaces. We apply the segmentation method to the Peppers image of Figure 1a using the same marked regions of Figure 8 but when the colour coordinates are in 3 different colourspaces: CIE LAB, HSV, and YCbCr. We test the RaWaCs algorithm also on the entire WBC dataset. The procedure consisted in the following steps:

- Mark the regions of interest;
- Consider the original image in a new colourspace;
- Apply the proposed procedure to the transformed image;
- Visualise the computed labels, obtained on the transformed image, on the original RGB image.

**Figure 11:**
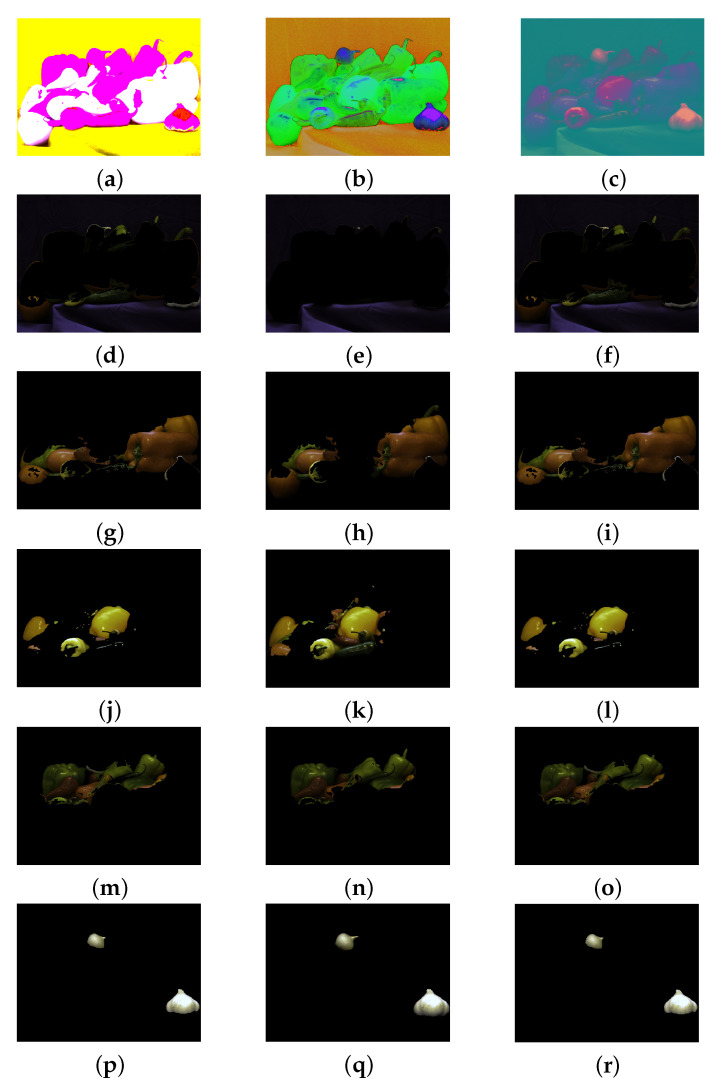
Performance comparison with respect to different colour spaces, Peppers image. (**a**–**c**) Peppers image in the CIELAB, HSV, YCbCr colourspaces, respectively. (**d**,**g**,**j**,**m**,**p**) segmentation result for CIELAB colourspace. (**e**,**h**,**k**,**n**,**q**) segmentation result for HSV colourspace. (**f**,**i**,**l**,**o**,**r**) segmentation result for YCbCr colourspace.

Figure 11 presents the segmentation results for the 3 different colourspaces mentioned before, obtained solving (16) with α=β=1. The proposed procedure reveals to be very robust with respect to the colourspaces: nonetheless, a visual inspection suggests that the HSV space seems to be the better choice, even with respect to the classical RGB space.

A deeper analysis is carried on the WBC dataset described in Section 3.2: 4 different colourspaces are considered and the RI, GCE, and ERR indexes are employed to assess the performance of the proposed strategy with respect to the chosen colourspace. Table 3 presents the results, showing that RaWaCs well performs wrt each colourspace, but one should note that the XYZ colourspace might be the most suitable choice for such images.

### 3.4. Adapting the Distance’s Weights

In the previous experiments, the employed similarity index is based on the Euclidean distance of the 8-neighbours of a pixel (see Figure 2) with respect to the centroids of the different labels. We note that this distance amounts to consider each pixel in the neighbourhood with the same weight. One possible strategy consists in weighting the informations of the neighbours with learned non-linear functions, using the information provided by the user-labelled regions. More precisely, a regression decision tree is fitted on the 8-neighbours of the labelled pixel to predict each dimension of the corresponding centroid. We use a regression tree, that searches for a greedy optimal binary recursive partitioning. In particular, we find a model that minimises holdout cross-validation loss: we employed the MatLab function fitrtree”, with hyperparameters automatically optimised. The fitted model is then used on the entire picture as a preprocess.

We apply this approach to the GrabCut dataset presented in Section 3.2: the obtained results are shown in Table 4. The performance on the GrabCut dataset is remarkable: an improvement of the 30.57% on Rand index and of 29.03% on the GCE index. This pre-processing procedure requires a large computational time, but on the other hand it helps in achieving better results for segmentation tasks for which the RaWaCs method was not designed for.

### 3.5. Biological Images

**Figure 12:**
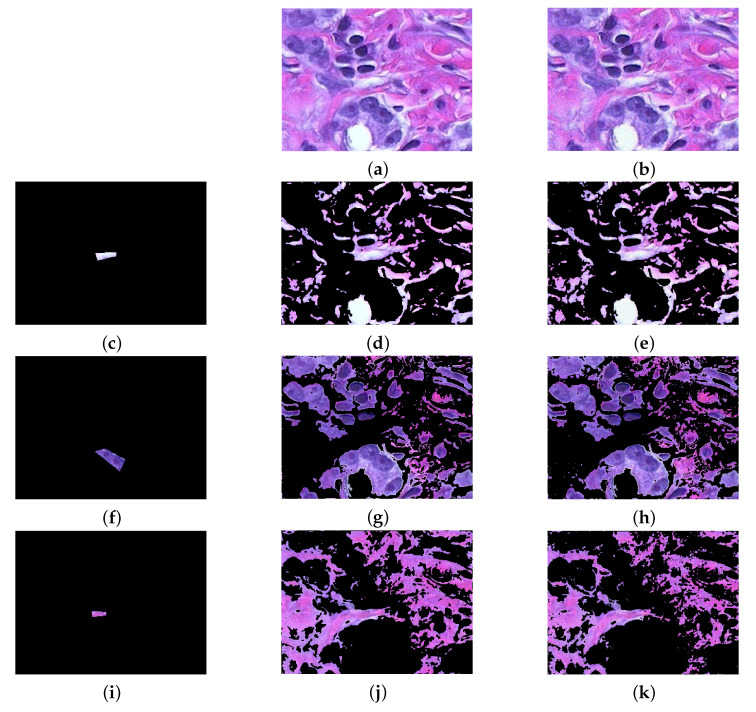
Matlab’s hestain image. (**a**) original image. (**b**) image affected by Poisson noise. (**c**) marked white background region, (**d**,**e**) segmentation of the white background on clean and noisy image, respectively. (**f**) marked region of the nuclei, (**g**,**h**) segmentation of the blue nuclei on clean and noisy image, respectively. (**i**) marked pink background region, (**j**,**k**) segmentation of the pink background on clean and noisy image, respectively. The results are obtained with α=1.2 for the clean image and with α=1.5 for the noisy image, while β is set to 1 in both cases.

**Figure 13:**
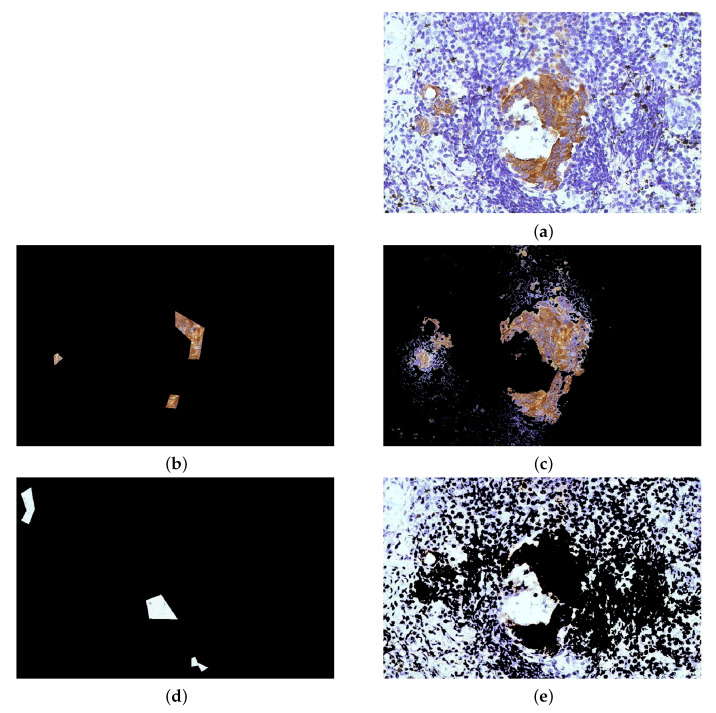
Matlab’s tissue image. (**a**) original image. (**b**) marked brown region, (**c**) segmentation results of the label. (**d**) marked background region, which includes both white background and blue cells. (**e**) segmentation of the background. The results are obtained with α=1.2,β=1.

We apply Algorithm 1 to several biological images. Figure 12a depicts the image of a tissue stained with hemotoxilyin and eosin and the relative segmentation results: the main aim consists in separating the blue nuclei form the background, whose main colours are white and pink. Figure 12b presents the same image affected by Poisson noise [58], added to each RGB channel with the MatLab function imnoise”. This type of statistical noise is common in electronic imaging, such as microscopy [2] and astronomy [59,60], due to the physics beyond the image acquisition process [46]. Figure 13 refers to an image of cells. In this case, the interest lies in separating the cell from the background and into distinguish the different cells. Both images are part of the Matlab’s Image Processing Toolbox, and they are both courtesy of Alan W. Partin, M.D., Ph.D., Johns Hopkins University School of Medicine.

The second column of Figure 12 shows the segmentation results obtained by setting α=1.2,β=1: the nuclei are well separated from the background, some regions of the pink tissue is included in the nuclei region due to the closeness with respect to the colour distance. When the noise is present, the influence of the similarity index must be increased, since the noise may alter the diffusion process. Suppose that two close but separated regions in the image have similar colours: since the Poisson noise is signal-dependent, it may alter the colour levels favouring, hence the diffusion between such regions, even in the case they are separated. On the other hand, the similarity index should be more robust with respect to the presence of the noise, since we are employing the centroids of the marked region. See Figure 3 and Equations (12) and (13). Note that this reasoning is valid when the noise level is low, i.e., when the pixels’ intensities are large. For a technical discussion about the dependence of the Poisson noise on the pixels values, the interested reader may see [46,61]. The third column in Figure 12 presents the segmentation results when this type of noise affects the image and when α is set to 1.5, while β=1 again. The performance of the proposed algorithm is still remarkable, even if the nuclei region includes a slightly larger amount of pink backgrounds.

Figure 13 presents more challenges with respect to Figure 12. The brown region is clearly distinguishable to the human eye, but its interior contains anyway several blue cells: this induces the procedure to include small regions with blue cells in the relative segmented part. This is clearly observable in the small marked region on the left of the image: the proposed procedure is able to explore the neighbourhoods and find other parts of interest which were not included in the original marking, but it also gathers inside this label several blue cells. This is due to the presence of this kind of corpuscles in the larger marked region on the right. In addition to this small amount of mislabelling, the performance is remarkable.

**Figure 14:**
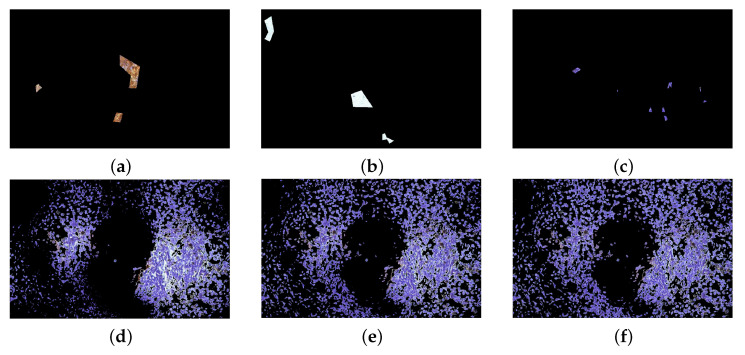
Comparison of the influence of the similarity index S in the final result. (**a**–**c**) marked regions of the brown part, of white background and blue cells, respectively. (**d**) α=1, (**e**) α=2, (**f**) α=3. The higher the value for α, the more precise the segmentation.

As previously observed, in some cases the colour distance plays a major role in RaWaCs approach: indeed, the objects in both Figure 12 and Figure 13 present very different colour. Consider the marked regions in the first row of Figure 14, which refer again to Figure 13a: these region refer to the brown part, the white background and the blue cells. When the interest lies in recognising the small blue corpuscles, the influence of the colour distance on the final result is evident: all the 3 different values for α provide reliable result, however a large value provides a slightly better result.

## 4. Conclusions

In this work we proposed an improvement of the random walker approach for semi-automatic segmentation. This is obtained by a new definition of similarity and distance between pixel using a given colour space and involving pixels in a neighbourhood. Then, a modified energy related to the random walker is considered coupling the probabilities of the RW and a global index as in classical clustering approach. The experimental results showed that the proposed approach is very robust with respect to the presence of noise and it overcomes more classical approaches, such as the *k*-means algorithm and the random walk method based on the pixel-wise Euclidean distance. Moreover, RaWaCs performs well on each colourspace, even if for particular classes of images (e.g., biological ones) a colourspace may be a more suitable choice than other ones. Furthermore, the proposed procedure has a remarkable performance wrt classical RW and more modern approaches, such as NRW and NLRW: indeed, RaWaCs is specially tailored for multi-labelling purposes and not only for foreground and background extraction, even if it performs well also in this latter task.

From the computational point of view, the most expensive steps concern the calculation of the new distance between pixels. However, we observe that these operations can be performed efficiently in parallel, for example with an appropriate implementation through the use of Graphics Processing Units (GPUs). Moreover, the computation of the probabilities of RW requires the numerical solution of linear systems which may be large, but sparse and well structured at the same time, consequently efficient algorithms can be used.

In the new method some hyper-parameters are to be fixed, see parameters α and β in Equation (16). In a future paper we will consider appropriate training and learning methods for the optimal choice of these parameters for some classes of images. Section 3.4 shows that an adaptive approach may help in learning suitable weights to be employed in the colour distance, paving the way to more sophisticated learning approaches. Furthermore, comparisons will be made with other semi-automatic methods, identifying suitable quality measures of the segmentation obtained.

## Figures and Tables

**Table 1:** Performance measures for RaWaCs, the random walk, the normalised random walk and the normalised lazy random walk methods. The former overcomes all the other methods. $\sigma_{g}$ is set to 90 and to 50 for NRW and for NLRW, respectively, while α=0.6 for NLRW (see [27] for the details). The average computational time is measured in seconds.

Method	RI	GCE	Err	Time
RaWaCs	0.9557	0.0598	0.0349	0.0252
RW	0.9312	0.0827	0.0526	0.0131
NRW	0.8838	0.1134	0.2317	0.0494
NLRW	0.8921	0.0998	0.2212	0.0501

**Table 2:** Performance measures for the proposed procedure, the random walk, the normalised random walk and the normalised lazy random walk methods on the GrabCut dataset. $\sigma_{g}$ is set to 90 for both NRW and NLRW, while α=0.9 for NLRW. The computational time is measured in seconds.

Method	RI	GCE	Err	Time
RaWaCs	0.9542	0.0427	0.0236	0.4734
RW	0.9499	0.0419	0.0277	0.2860
NRW	0.9493	0.0410	0.2428	8.0130
NLRW	0.9575	0.0361	0.2375	8.7822

**Table 3:** Performance measures for the proposed procedure with respect to the chosen colourspace. The performance measurements are the same described in Section 3.2. The computational time is measured in seconds.

Colour Space	RI	GCE	Err	Time
RGB	0.9557	0.0598	0.0349	0.0252
LAB	0.9524	0.0610	0.0363	0.0248
XYZ	0.9631	0.0598	0.0353	0.0260
YCbCr	0.9566	0.0580	0.0338	0.0256

**Table 4:** Performance measures for the proposed procedure coupled with a pre-processing procedure based on machine learning technique. The performance of the RawaCs algorithm improves wrt to each evaluation index: the sole drawback is the high computational time required. The computational time is measured in seconds.

Dataset	RI	GCE	Err	Time
GrabCut	0.9682	0.0303	0.0163	463.20

## Data Availability

The code for the MatLab implementation of RaWaCs is available at https://github.com/AleBenfe/RaWaCs. Publicly available datasets were analyzed in this study. This data can be found here: https://github.com/zxaoyou/segmentation_WBC (WBC dataset) and https://www.robots.ox.ac.uk/~vgg/data/iseg/ (GrabCut dataset).
